# Community pharmacists’ perspectives on cardiovascular disease pharmaceutical care in the United Arab Emirates: a questionnaire survey-based analysis

**DOI:** 10.3389/fphar.2023.1237717

**Published:** 2023-09-05

**Authors:** Ammar Abdulrahman Jairoun, Sabaa Saleh Al-Himyari, Moyad Shahwan, Mina Al Ani, Mustafa Habeb, Maimona Jairoun, Sa’ed H. Zyoud, Mustfa Faisal Alkhanani, Reem Hasaballah Alhasani, Alqassem Y. Hakami, Bayan A. Ainousah, Fahad S. Alshehri, Nasser M. Alorfi, Abdulhaq Suliman

**Affiliations:** ^1^ Health and Safety Department, Dubai Municipality, Dubai, United Arab Emirates; ^2^ Discipline of Clinical Pharmacy, School of Pharmaceutical Sciences, University of Science Malaysia (USM), Pulau Pinang, Malaysia; ^3^ Pharmacy Department, Emirates Health Services, Dubai, United Arab Emirates; ^4^ Department of Clinical Sciences, College of Pharmacy and Health Sciences, Ajman University, Ajman, United Arab Emirates; ^5^ Centre of Medical and Bio-Allied Health Sciences Research, Ajman University, Ajman, United Arab Emirates; ^6^ Department of Developmental Biology and Cancer, Great Ormond Street Institute of Child Health, Faculty of Population Health Sciences, University College London, London, United Kingdom; ^7^ Edgware Community Hospital, Edgware, United Kingdom; ^8^ Department of Clinical and Community Pharmacy, College of Medicine and Health Sciences, An-Najah National University, Nablus, Palestine; ^9^ Clinical Research Centre, An-Najah National University Hospital, Nablus, Palestine; ^10^ Department of Biology, College of Science, University of Hafr Al Batin, Hafr Al-Batin, Saudi Arabia; ^11^ Department of Biology, College of Applied Science, Umm Al-Qura University, Makkah, Saudi Arabia; ^12^ College of Medicine, King Saud bin Abdulaziz University for Health Sciences, Jeddah, Saudi Arabia; ^13^ Pharmaceutical Chemistry Department, College of Pharmacy, Umm Al Qura University, Makkah, Saudi Arabia; ^14^ Department of Pharmacology and Toxicology, College of Pharmacy, Umm Al Qura University, Mecca, Saudi Arabia; ^15^ College of Dentistry, Ajman University, Ajman, United Arab Emirates

**Keywords:** community pharmacists, cardiovascular disease, blood pressure, patient screening, cardiovascular disease management

## Abstract

**Background:** Community pharmacists play an intermediary role between prescribing physicians and patients in the United Arab Emirates (UAE) and thus are responsible for ensuring that patients receive optimal cardiovascular disease (CVD) pharmaceutical care.

**Methods:** we used a cross-sectional design to assess the perceptions and practices of community pharmacists concerning pharmaceutical care for patients with CVD. A trained researcher visited randomly selected community pharmacies and used a structured questionnaire to conduct in-person interviews with pharmacists. The questionnaire collected demographic data and information on perceptions and practices regarding CVD pharmaceutical care.

**Results:** Five hundred and fifty-one participants were recruited. The average participant age (mean ± SD) was 35 ± 2.7 years. The average perception score regarding CVD prevention and management was 75.6% (95% confidence interval [CI] 77.1%–74.2%), and the average practice score for CVD prevention and management was 87.1% (95% CI 76.5%–79.6%). Bivariate analysis revealed that gender (*p* = 0.001), education level (*p* < 0.001), pharmacy position (*p* = 0.004), work experience (*p* < 0.001), number of patients served per day (*p* < 0.001) and being trained on CVD prevention and management (*p* < 0.001) were significantly associated with perceptions about the prevention and management of CVD. Better practice scores were seen among older participants (OR 1.01; 95% CI 1–1.019), postgraduates (OR 1.77; 95% CI 1.66–1.89), workers at chain pharmacies (OR 1.24; 95% CI 1.11–1.39), pharmacists in charge (OR 1.22; 95% CI 1.01–1.47), pharmacists with >10 years of experience (OR 11.3; 95% CI 6.01–15.62), pharmacists with 6–10 years of experience (OR 4.42; 95% CI 3.90–5) and pharmacists trained on CVD prevention and management (OR 1.29; 95% CI 1.15–1.46).

**Conclusion:** Pharmacy practitioners working in community pharmacies in the UAE actively engage in delivering pharmaceutical care to patients, playing a role in CVD management and prevention. However, they showed low levels of involvement in other healthcare services, specifically in screening and measuring patients’ weight, glucose levels, and blood pressure, monitoring treatment responses, maintaining medical records, and reviewing medication refill histories. Activities such as educating patients, providing medication counseling, offering support for treatment adherence, and fostering collaborative relationships with other healthcare providers should be encouraged among UAE community pharmacists to ensure the provision of high-quality patient care.

## 1 Introduction

Cardiovascular disease (CVD) is the leading contributor to the global burden of disease (GBD, 2015 Risk Factors Collaborators, 2016). The incidence of CVD in developed countries has notably declined over recent decades. However, this decline has recently plateaued. Some middle-income countries have experienced a decline in CVD mortality rates, while most developing countries have reported a rapid increase in incidence (Mensah et al., 2019). In 2015, there were 422.7 million recorded cases of CVD, ischemic heart disease, and stroke, which were the leading causes of death (Mensah et al., 2019). It has been estimated that over 17 million individuals die annually. In 2019, CVD was the main cause of almost one-third of all deaths in adults under the age of 70 (Roth et al., 2020). Regarding the accessibility and availability of routine health checks, previous research reported false health beliefs and poor awareness, and limited access to routine health checks in primary healthcare settings in most Arabic countries, highlighting the need for more proactive and regular health screening (Alorfi and Ashour, 2022; Alorfi et al., 2023a; Alorfi et al., 2023b). Pharmacists delivering health screenings could reduce the financial and psychological barrier for patients who face difficulties in accessing primary healthcare (Ayorinde et al., 2013). Regarding feasibility, pharmacist-delivered basic screening for a variety of conditions, including CVD, could reduce the pressure on primary healthcare services and enable the early diagnosis of CVD (San-Juan-Rodriguez et al., 2018).

Community pharmacists have frequent face-to-face contact with around 90% of the population yearly, particularly with those at an elevated risk of developing CVD (Alzubaidi et al., 2018). There are an estimated 2500 licensed community pharmacies in the UAE that are open daily for an average of 13 h (Alzubaidi et al., 2018). Therefore, there is significant potential for pharmacists to participate in promoting public health awareness and play a significant role in CVD prevention, as they are among the most visited healthcare professionals and often serve as the first lines of contact for individuals. In addition to dispensing medications, pharmacists can offer more direct interventions involving medication and disease management education to support doctors’ efforts to increase medication adherence, achieve desired therapeutic outcomes, and improve safe medication use (George et al., 2010; Omboni and Caserini, 2018).

Community pharmacists can significantly enhance patient care by providing chronic disease management options (Bacci et al., 2019; Alhazmi et al., 2022). Community pharmacies are the first healthcare facilities where patients may receive prescriptions and treatments at the community level. As it is hard for many patients to locate primary care doctors, and healthcare costs are rapidly rising, community-based care models are being promoted (George et al., 2010). Community pharmacists may significantly assist in enhancing health outcomes by minimizing drug-related problems and encouraging medication adherence, which reduces the need for unnecessary doctor visits (George et al., 2010). Currently, there is no systematic CVD screening program within the primary healthcare setting in the UAE (Alzubaidi et al., 2019), leaving a significant proportion of CVD untreated and undiagnosed.

In the UAE healthcare system, community pharmacists play a vital role by focusing primarily on delivering pharmaceutical care and medication management services to their respective communities. Unlike hospital pharmacists who operate within medical facilities, community pharmacists are at the forefront of patient care within their local community. They fulfill their responsibilities in retail pharmacies, actively collaborating with physicians and other healthcare professionals to ensure safe and effective use of medications.

The educational prerequisites for community pharmacists in the UAE are comparable to those in other countries. To obtain a license as a community pharmacist in the UAE, individuals must successfully complete a Bachelor of Pharmacy (BPharm) degree from an accredited institution, which typically takes 4–5 years. The degree program encompasses a wide range of subjects, including pharmaceutical sciences, clinical pharmacy, pharmacology, and pharmacy practice. Following graduation, aspiring pharmacists are required to undergo an internship or training program sanctioned by the Ministry of Health and Prevention (MOHAP) in the UAE. This program equips pharmacists with practical experience in community pharmacy settings, ensuring that they acquire the necessary skills for safe and effective practice.

Individuals aspiring to practice as community pharmacists in the UAE must obtain a professional license from the MOHAP. The licensing procedure involves successfully completing a qualifying examination that assesses a candidate’s knowledge and competence across various aspects of pharmacy practice. The examination covers subjects such as pharmacology, therapeutics, drug dispensing, pharmaceutical calculations, and professional ethics. Community pharmacists in the UAE devote themselves to providing accessible and patient-centered care, fostering medication safety, and enhancing health outcomes within the local community they serve. Continuous training and adherence to guidelines can benefit community pharmacists, assisting them in their professional pursuits, such as screening, lifestyle modification, prevention, and treatment strategies for CVD. These advantages can extend to include rural populations as well. The aim of this study is to assess the perceptions and practices of community pharmacists concerning pharmaceutical care for patients with CVD in the UAE.

## 2 Methods and materials

### 2.1 Study setting and design

This study used a cross-sectional design to assess the perceptions and practices of community pharmacists regarding pharmaceutical care for patients with CVD. Random sampling was used to select UAE community pharmacies for inclusion. Seven trained final-year pharmacy students went to the selected pharmacies between December 2022 and March 2023 and performed in-person interviews with the pharmacists. The student interviewers underwent thorough interview training, which involved familiarization with the scientific terminology used and how the questionnaire should be applied. Our prior experience has demonstrated that this step is necessary to improve interviewers’ skills and reduce the survey error rate.

### 2.2 Definition of pharmaceutical care for patients with CVD

Delivering pharmaceutical care to individuals diagnosed with CVD involves collaborative efforts between pharmacists and other healthcare professionals to ensure personalized care, effective medication management, and patient education. Pharmacists play a fundamental role in evaluating patients’ medication regimens, selecting the appropriate dosages, and promoting adherence to the prescribed treatments. Additionally, they monitor patients for potential drug interactions and adverse effects while offering guidance on lifestyle modifications such as diet, exercise, and smoking cessation.

### 2.3 Research instrument development

A structured questionnaire was developed based on an initial literature review (George et al., 2010; Alzubaidi et al., 2018; Omboni and Caserini, 2018; Alzubaidi et al., 2019) and modified to suit the UAE context while retaining its main key points. Opinions from experts in the cardiology and rheumatic heart disease fields were sought to ensure the suitability of the questionnaire design and its relevance to the current work. The instrument was further evaluated by six members of the Faculty of Medicine and Clinical Pharmacy at Ajman University to ensure it was appropriate and relevant. Some minor adjustments were made based on the experts’ recommendations, such as defining the scientific terminology, changing the question and page numbering, using the field name of Gender rather than Sex for all questions, linking certain questions, and concluding the questionnaire with a specific point.

Before the pilot testing was conducted, the content validity of the instrument was tested using Lawshe’s content validity (Lawshe, 1975), in which items with a content validity ratio (CVR) of above 0.78 are considered acceptable, while any items beneath this threshold are removed. All questionnaire items had CVR values over 0.78; therefore, they demonstrated acceptable validity. The content validity index (CVI) of the final instrument was subsequently calculated using means of the acceptable items. The questionnaire had a CVI value of 0.891, indicating acceptable overall content validity (Kwon and Kim, 2003).

The instrument’s face validity was assessed through pilot testing, which involved 25 community pharmacists and took place between December 18–30, 2022. The data gained during this phase was not used in the final analysis. The questionnaire was completed successfully by 21 of the participants. The results were used to evaluate the questionnaire’s reliability and estimate the ideal sample size for the main study. The reliability was estimated using Cronbach’s α. The questionnaire had a Cronbach’s α of 0.77, indicating acceptable internal reliability.

### 2.4 Research instrument sections

The questionnaire comprised three sections:

• Part 1—Eight items assessing respondents’ demographic information, including gender, position in the pharmacy (pharmacist in charge/chief pharmacist/assistant pharmacist); assistant pharmacist is a pharmacy technician or pharmacy assistant, is a healthcare professional who supports the work of pharmacists in various pharmacy settings. They work under the supervision of a registered pharmacist. Work experience in years, hours worked per day, number of patients seen per day, and any training received on CVD prevention and management. This training could involve education on assessing patients’ medication regimens, ensuring appropriate drug selection and dosages, and ensuring adherence to prescribed therapies.
• Part 2—Eleven items assessing respondents’ perceptions of CVD pharmaceutical care.
• Part 3—Eighteen items assessing respondents’ practices regarding CVD pharmaceutical care.

### 2.5 Questionnaire scoring

The questionnaire used 11 items to assess respondents’ perceptions of CVD pharmaceutical care. Respondents could answer each item with “Agree” or “Disagree,” with each item having one correct answer. Each correct answer scored one point, and the points were summed to give the total perception score.

Eighteen items were used to evaluate the respondents’ practices regarding CVD pharmaceutical care. Participants used a 5-point Likert scale to rate the items (1 = “Not at all involved,” 2 = “Somewhat involved,” 3 = “Uncertain,” 4 = “Involved,” and 5 = “Very involved”). A “very involved” response was considered to represent good practice. These responses scored one point. All other options were considered to be negative practice and received no points. The points were summed to produce the overall practice score.

Good perception and good practice scores were determined by calculating the respective median scores, allowing the respondents’ scores to be classified as “good perception” and “good practice.”

• The median perception score was 9. Therefore, any respondents who scored at least 9 were considered to have good perception. Those who scored below 9 were considered to have poor perception.
• The median perception score was 15. Therefore, any respondents who scored at least 15 were considered to have good practice. Those who scored below 15 were considered to have poor practice.

### 2.6 Sample size estimation

The pilot study results were used to calculate the ideal sample size for the main study. The overall response rate in the pilot phase was 84%. The pilot participants were asked to answer the item, “Do you provide and practice pharmaceutical care for CVD?” Just over half (60%) responded that they did. The 5% alpha level used meant that the confidence interval (CI) was set to 95%. With precision (D) of 5%, the maximum width of the 95% CI was 10%. Therefore, assuming that the main study would have a similar non-response rate of 40%, the ideal sample size was calculated to be 615 participants. Situations can occur wherein the sample size actually achieved is not enough to meet the sample size as calculated in advance. Such an outcome may be due to several factors (Supplementary Material)

### 2.7 Target population

Specific criteria were used to select the sample for the main study. The inclusion criteria were community pharmacists who had a minimum of 3 months of experience working in independent pharmacies or chain pharmacies registered with a relevant authority (i.e., the Ministry of Health, the Dubai Health Authority, or the Health Authority Abu Dhabi). The exclusion criteria were pharmacists with less than 3 months of experience working in a pharmacy, those who were only recently qualified as pharmacists or on a probationary period, and those working in pharmacies not registered with any of the abovementioned health authorities.

### 2.8 Sampling technique

A survey conducted in the UAE in 2010 revealed that there were 2000 active community pharmacies (Jairoun et al., 2022). To ensure adequate representation of the pharmacies in this research, the stratified random sampling technique was employed. First, the location and contact details of community pharmacies located in the study region were obtained from local business directories and the Yellow Pages. Then, all active community pharmacies were stratified into groups according to their location. This produced three strata: community pharmacies in Abu Dhabi, community pharmacies in Dubai, and community pharmacies in the Northern Emirates.

After the community pharmacies had been stratified, their relevant data were entered into the sampling frame in an Excel spreadsheet. The data entered included name, type, location, and contact details (phone number and email address). Then, each community pharmacy was assigned a unique ID number. Following this, 615 community pharmacies were chosen based on a simple random selection of the listed ID numbers. The chosen community pharmacies were then sorted by type and location.

### 2.9 Data collection

Trained researchers visited the selected pharmacies between 1 January 2023 and 25 March 2023. The pharmacists were informed of the research purpose and asked to provide their email addresses. The researchers then used the structured questionnaire to conduct in-person interviews with the pharmacists.

### 2.10 Statistical analysis

We used SPSS Version 26 for the data analysis. Frequencies and percentages were used to examine the categorical variables, while means and standard deviations (SD) were used to assess the continuous, normally distributed quantitative variables. Unpaired student t-tests, one-way ANOVAs, and non-parametric variants were used to identify differences between the quantitative variables. A Shapiro-Wilk test was used to evaluate normality. Normally distributed continuous variables were indicated by *p* > 0.05. A normal Q-Q Plot was visually examined. Multivariate logistic regression models were used to identify the factors affecting the perception and practice of community pharmacists regarding CVD. Statistical significance was assumed when *p* > 0.05.

### 2.11 Ethical considerations

The Institutional Ethical Review Committee of Ajman University granted approval for this study. All respondents were informed about the study’s purpose prior to data collection and agreed that the questionnaire could only be completed with their full consent. All respondents provided written and informed consent. No details were collected from the participants that could reveal their identities, and care was taken to maintain participant confidentiality.

## 3 Results

### 3.1 Participant demographic and baseline characteristics

Five hundred and fifty-one participants were recruited. The response rate was 89.6%. The average participant age was 35 ± 2.7 years. Most participants were female (61.9%), and most had a Bachelor’s degree (84.4%). Of the pharmacies included, 51% (*n* = 81) were independent pharmacies, and 49% (*n* = 270) were chain pharmacies. Most participants were the pharmacists in charge (70.6%), and 72.1% had 6–10 years of experience. Approximately 69.7% (*n* = 384) worked >8 h/day, and 30.3% (*n* = 167) worked ≤8 h/day. Regarding the number of patients served per day, 20% of pharmacists (*n* = 110) served 5–10, 58.3% (*n* = 321) served 11–20, and 21.8% (*n* = 120) served >20. Overall 56.4% (*n* = 311) of participants had received CVD prevention and management training (Table 1).

**TABLE 1:** Participant demographic information (*n* = 551).

Demographic variable	Group	Frequency	Percentage (%)
Gender	Male	210	38.1
	Female	341	61.9
Educational level	Bachelor’s degree	465	84.4
	Postgraduate	86	15.6
Pharmacy type	Independent pharmacy	281	51
	Chain pharmacy	270	49
Position in the pharmacy	Pharmacist in charge	389	70.6
	Chief pharmacist	88	16
	Assistant pharmacist	74	13.4
Work experience	1–5 years	75	13.6
	6–10 years	397	72.1
	> 10 years	79	14.3
Hours worked/day	≤ 8 h	167	30.3
	> 8 h	384	69.7
Numbers of patients served per day	5–10	110	20
	11–20	321	58.3
	> 20	120	21.8
Received CVD prevention and management training	Yes	311	56.4
	No	240	43.6

Note: postgraduate refers to a Master’s degree or Ph.D., and an assistant pharmacist is a healthcare professional who supports the work of pharmacists in various pharmacy settings, working under the supervision of a registered pharmacist.

### 3.2 The role of perceptions in CVD prevention and management within community pharmacies

The average perception score regarding CVD prevention and management was 75.6% (95% CI 74.2%–77.1%). The average practice score regarding CVD prevention and management was 87.1% (95% CI 76.5%–79.6%). In general, the perceptions and practices regarding CVD prevention and management among the participants were good. The results of each question related to perception and practice are shown in Tables 2, 3 **.**

**TABLE 2:** Participants’ perceptions regarding CVD prevention and management.

Perception item	Disagree		Agree	
	F	%	F	%
1. Cardiovascular disease is a common and increasingly prevalent condition in the UAE	21	3.8	530	96.2
2. A sedentary lifestyle and obesity are the primary causes of CVD	60	10.9	491	89.1
3. There should be more attention to modifying the general population’s lifestyles	149	27	402	73
4. It is important to identify patients with CVD to address the multiple risk factors involved	38	6.9	513	93.1
5. The risks of CVD can be reduced by following a weight loss program	63	11.4	488	88.6
6. The risks of CVD can be decreased through increased exercise	67	12.2	484	87.8
7. Drugs should be prescribed to treat the individual aspects of CVD	70	12.7	481	87.3
8. The risks of CVD can be reduced through the use of dietary and herbal supplements	307	55.7	244	44.3
9. The risks of CVD can be reduced by restricting alcohol consumption	74	13.4	477	86.6
10. Patients should be screened at an early stage to avoid severe outcomes	24	4.4	527	95.6
11. When evaluating CVD, it is important to document the patient’s drug refill history	53	9.6	498	90.4

Abbreviations: CVD, cardiovascular disease; F, frequency; UAE, UUnited Arab Emirates; %, Percentage.

**TABLE 3:** Participants’ practices regarding CVD prevention and management.

Practice item	Not at all involved		Little involved		Uncertain		Involved		Very involved	
	F	%	F	%	F	%	F	%	F	%
1. Advise patients to lose weight by following a low-calorie diet	43	7.8	62	11.3	109	19.8	107	19.4	230	41.7
2. Promote physical exercise	81	14.7	27	4.9	70	12.7	161	29.2	212	38.5
3. Advise patients to restrict their salt intake	43	7.8	43	7.8	90	16.3	147	26.7	228	41.4
4. Provide counseling on which diets lower cholesterol (i.e., reduced intake of cholesterol/saturated fats)	46	8.3	31	5.6	96	17.4	227	41.2	151	27.4
5. Advise patients to follow a vegetable-rich diet	12	2.2	25	4.5	97	17.6	150	27.2	267	48.5
6. Advise patients to increase the amount of soluble fiber in their diet	34	6.2	16	2.9	99	18.0	145	26.3	257	46.6
7. Provide counseling on exercising caution when choosing over-the-counter drugs or herbal products, as these have been linked to an increased risk of cardiovascular disease	43	7.8	62	11.3	109	19.8	107	19.4	230	41.7
8. Advise patients to limit their alcohol consumption	34	6.2	83	15.1	117	21.2	163	29.6	154	27.9
9. Advise patients to quit smoking	36	6.5	41	7.4	112	20.3	87	15.8	275	49.9
10. Review the drug refill history of the patient to assess whether they are following the therapy	324	58.8	169	30.7	50	9.1	8	1.5	0	0
11. Provide counseling on why patients should routinely monitor their weight, blood glucose, and blood pressure, as well as why they should achieve their health targets	46	8.3	31	5.6	96	17.4	227	41.2	151	27.4
12. Offer patients screening and measurement services for weight, blood glucose, and blood pressure	471	85.5	56	10.2	20	3.6	4	0.7	0	0
13. Sell blood pressure and blood glucose home monitoring equipment	90	16.3	21	3.8	41	7.4	56	10.2	343	62.3
14. Advise patients on how prescription treatment and self-care can help mitigate the components of CVD	43	7.8	62	11.3	109	19.8	107	19.4	230	41.7
15. Encourage patients to follow the treatment	16	2.9	13	2.4	66	12.0	107	19.4	349	63.3
16. Monitor how the patients respond to the treatment	261	47.4	197	35.8	81	14.7	6	1.1	6	1.1
17. Maintain patient care service records in the pharmacy	374	67.9	122	22.1	44	8.0	11	2.0	0	0
18. If necessary, refer patients to a doctor	274	49.7	199	36.1	68	12.3	8	1.5	2	0.4

Abbreviations: CVD, cardiovascular disease; F, frequency; %, Percentage.

The bivariate analysis revealed that gender (*p* = 0.001), education level (*p* < 0.001), position in the pharmacy (*p* = 0.004), work experience (*p* < 0.001), number of patients served per day (*p* < 0.001) and having received training on CVD prevention and management (*p* < 0.001) were significantly associated with perceptions regarding CVD prevention and management among the participants (Table 4).

**TABLE 4:** Comparison of participants’ perceptions and practices regarding CVD across different demographic variable.

Demographic variable	Perception score (11 items)				Practice score (18 items)			
	Mean	95% CI		P-value	Mean	95% CI		P-value
**Gender**								
Male	70.69	68.16	73.22	0.001*	75.87	73.12	78.63	0.001*
Female	78.67	77.04	80.29		79.39	77.45	81.33	
**Education level**								
Bachelor	74.15	72.63	75.68	<0.001*	74.16	68.99	79.33	0.04*
Postgraduate	83.62	80.04	87.19		78.77	77.13	80.40	
**Pharmacy type**								
Independent pharmacy	74.28	72.22	76.34	0.058	73.72	71.37	76.07	<0.001*
Chain pharmacy	77.04	75.07	79		82.55	80.52	84.59	
**Position in pharmacy**								
Pharmacist in charge	76.47	74.87	78.06	0.004*	80.96	79.17	82.76	<0.001*
Chief pharmacist	77.06	73.83	80.30		71.34	67.24	75.44	
Assistant pharmacist	69.53	64.33	74.73		70.72	65.95	75.49	
**Work experience**								
1–5 years	46.42	44.37	48.47	<0.001*	61.48	57.15	65.82	<0.001*
6–10 years	76.85	75.73	77.97		80.66	78.92	82.41	
> 10 years	97.24	96.29	98.18		80.66	76.53	84.79	
**Number of patients served per day**								
5–10	78.02	75.04	80.99	<0.001*	98.33	97.64	99.02	<0.001*
11–20	78.67	77.10	80.25		82.21	81.23	83.19	
> 20	65.30	61.56	69.04		48.33	46.19	50.47	
**Hours worked/day**								
≤ 8 h	73.71	70.99	76.42	0.081	72.75	70.32	75.19	0.001*
> 8 h	76.47	74.79	78.14		80.35	78.36	82.35	
**Received CVD prevention and management training**								
Yes	87.66	86.65	88.68	<0.001*	75.05	72.36	77.73	0.001*
No	60.04	58.59	61.49		80.37	78.47	82.27	

Notes: *p < 0.05 was considered statistically significant. P-values were calculated using an independent *t*-test and one-way ANOVA., Postgraduate refers to a Master’s degree or Ph.D., and an assistant pharmacist is a healthcare professional who supports the work of pharmacists in various pharmacy settings, working under the supervision of a registered pharmacist.

Abbreviations: CI, confidence interval; CVD, cardiovascular disease.

Similarly, there were statistically significant relationships between CVD prevention and management practice and gender (*p* < 0.001), education level (*p* = 0.04), pharmacy type (*p* < 0.001), position in the pharmacy (*p* < 0.001), work experience (*p* < 0.001), number of patients served per day (*p* < 0.001), working hours per day ((*p* = 0.001) and have received training on CVD prevention and management (*p* = 0.001) (Table 4).

### 3.3 Determining factors in the perceptions and practices for CVD management among community pharmacists

Better perception scores were observed among female pharmacists (OR 1.19; 95% CI 1.03–1.37), chief pharmacists (OR 1.45; 95% CI 1.55–1.82), pharmacists in charge (OR 1.24; 95% CI 1.03–1.49), pharmacists with >10 years of experience (OR 7.44; 95% CI 3.61–9.83), pharmacists with 6–10 years of experience (OR 2.25; 95% CI 1.85–2.74), pharmacists who served 5–10 patients/day (OR 1.37; 95% CI 1.10–1.70), and pharmacists trained on CVD prevention and management (OR 2.69; 95% CI 2.32–3.14) (Table 5).

**TABLE 5:** Multivariate regression analysis of the factors associated with perceptions and practices regarding CVD prevention and management.

	Good perception ≥9				Good practice ≥15			
Demographic variable	OR	95% CI		P-value	OR	95% CI		P-value
**Gender (Ref. Male)**								
Female	1.19	1.03	1.37	0.022*	1.06	0.93	1.21	0.411
**Education level (Ref. Bachelor’s degree)**								
Postgraduate degree	0.95	0.77	1.16	0.613	1.77	1.66	1.89	0.001*
**Pharmacy type (Ref. Independent pharmacy)**								
Chain Pharmacy	1.09	0.94	1.25	0.261	1.24	1.11	1.39	0.001*
**Position in the pharmacy (Ref. Assistant pharmacist)**								
Chief pharmacist	1.45	1.155	1.82	0.001*	1.87	0.75	1.067	0.712
Pharmacist in charge	1.24	1.034	1.49	0.021*	1.22	1.01	1.468	0.038*
**Work experience (Ref. 1–5 years)**								
>10 years	7.44	3.61	9.83	<0.001*	11.3	6.01	15.62	<0.001*
6–10 years	2.25	1.85	2.74	<0.001*	4.42	3.90	5.00	<0.001*
**Number of patients served daily (Ref. > 20)**								
5–10	1.37	1.10	1.70	0.004*	1.09	0.85	1.42	0.47
1–20	1.12	0.94	1.33	0.213	0.97	0.82	1.17	0.79
**Working hours/day (Ref. >8 h)**								
≤ 8 h	0.99	0.86	1.15	0.943	1.03	0.91	1.15	0.667
**Received CVD prevention and management training (Ref. No)**								
Yes	2.69	2.32	3.14	<0.001*	1.29	1.15	1.46	<0.001*
Age	1.003	0.99	1.02	0.589	1.010	1.000	1.019	0.044*

Notes: *p < 0.05 was considered statistically significant, and the good knowledge and practice scores were determined based on the median scores. Postgraduate refers to a Master’s degree or Ph.D., and an assistant pharmacist is a healthcare professional who supports the work of pharmacists in various pharmacy settings, working under the supervision of a registered pharmacist.

Abbreviations: CVD, cardiovascular disease.

Better practice scores were observed among older pharmacists (OR 1.01; 95% CI 11.019), pharmacists with postgraduate education (OR 1.77; 95% CI 1.66–1.89), pharmacists who worked at chain pharmacies (OR 1.24; 95% CI 1.11–1.39), pharmacists in charge (OR 1.22; 95% CI 1.01–1.47), pharmacists with >10 years of experience (OR 11.3; 95% CI 6.01–15.62), pharmacists with 6–10 years of experience (OR 4.42; 95% CI 3.90–5) and pharmacists trained on CVD prevention and management (OR 1.29; 95% CI 1.15–1.46) (Table 5).

### 3.4 Barriers and obstacles to CVD-related counseling and health promotion services in community pharmacies

A lack of coordination with other healthcare providers (85.6%), lack of time (75%), lack of knowledge or clinical skills (71%), and patients being uninterested in preventive activities (56%) were the most commonly cited barriers to providing counseling and health promotion services for CVD in community pharmacies (Table 6).

**TABLE 6:** Barriers to CVD-associated counseling and health promotion services in community pharmacies (*n* = 551).

Obstacles and barriers	Frequency	Percentage (%)
Lack of private counseling area	209	38
Lack of time	413	75
Workload and/or shortage of pharmacy staff	237	43
Lack of educational materials	116	21
Lack of knowledge and skills	391	71
Lack of coordination with other healthcare professionals	472	85.6
Patients being uninterested in preventive activities	309	56
Lack of clinical tools	176	32

## 4 Discussion

For CVD patients, community pharmacies are outpatient healthcare services that they can easily access to receive medication and other care services. Therefore, community pharmacists play an intermediary role between prescribing physicians and patients and are responsible for ensuring that patients receive the correct medications. This is a critical service, and these pharmacists are well-positioned to prevent prescription errors and perform additional checks to ensure that the medication is safe for use (Hasan et al., 2012; Alzubaidi et al., 2019). This study sought to assess the level of CVD pharmaceutical care offered by community pharmacies located in the UAE. The findings revealed good perceptions and practices on CVD prevention and management among community pharmacists. However, there is still a need to improve their involvement in certain aspects of patient care. Specifically, the care services offered to patients by community pharmacists could be improved by measuring patients’ weight and glucose and blood pressure levels, reviewing their medication refill history, monitoring their treatment response, referring them to a physician when needed, and maintaining patient records.

The participants in this study demonstrated good perception levels and good involvement in providing CVD pharmaceutical care, with an average perception score of 75.6%. Therefore, the participants held generally positive perceptions of the care services for CVD patients, particularly concerning the need to reduce the patient risk, inform patients of prevention methods, and provide appropriate treatment approaches. This is in line with prior research conducted in Malaysia, which reported good perception levels concerning CVD prevention, management, and risks (Sia et al., 2020).

Most of the participants in the current study agreed that CVD is a common condition, and its rates have been rising in the UAE, with 89.1% of participants expressing that this increase is being driven by the population’s sedentary lifestyle and increasing obesity rates. This is in line with research in Kuwait and in the USA, where community pharmacists agreed that chronic heart conditions are an urgent public health challenge (McKenney et al., 2004). A substantial proportion of participants in the current study stated that modifying patient lifestyles should be given more attention.

A large proportion of the participants in the current study (>85%) agreed that CVD risks could be reduced by maintaining a healthy weight, consuming less alcohol, and engaging in more physical exercise. These findings are similar to those of prior research (Erku and Mersha, 2017; Belachew et al., 2020). The current results indicated that the participants had considerable awareness of CVD, its common risks, and treatment priorities, and held attitudes towards CVD. This supports prior research in Lebanon, which reported a consensus among community pharmacists regarding the importance of weight reduction in this population (Hijazi et al., 2020; Sulaiteen et al., 2023). The high knowledge levels observed among the current participants provide a solid foundation for conducting feasibility research regarding using community pharmacies to effectively perform patient screening for CVD in the UAE.

The current results are encouraging, as they indicate that most of the participants were well involved in CVD management. A substantial number of participants reported providing advice to patients on ways to reduce weight, such as consuming fewer calories (61.2%), regulating their consumption of salt and alcohol (68%), engaging in physical exercise (67.7%), and not smoking (65.7%). This is in line with previous research conducted in several contexts (George et al., 2011; Puspitasari et al., 2013; Fonseca et al., 2021), which found that community pharmacists are often engaged in CVD prevention and promote healthy lifestyles among their patients. In the current study, a greater proportion of participants provided advice regarding restricting the consumption of alcohol and ceasing smoking than in other studies (Laliberte et al., 2012; Sia et al., 2020). This indicates that the increase in sedentary lifestyles has caught the attention of pharmacy professionals, causing them to become more involved in providing advice on the risks of alcohol and smoking.

Only a fraction (<5%) of the participants surveyed in this study reported being involved in other practices, such as screening, measuring patients’ weight, glucose levels, and blood pressure monitoring treatment responses, maintaining medical records, and reviewing individual medication refill histories. This reduced involvement implies that the participants do not provide patient counseling on all CVD aspects and is in line with a prior study (Belachew et al., 2020). The level of involvement in these practices reported in the current study was significantly lower than that reported in Malaysia (Sia et al., 2020) and Kuwait (Katoue et al., 2013). In addition, the proportion of participants in the current study who reported that they monitored their patients’ treatment responses was lower than in Kuwait (Katoue et al., 2013). These conflicting findings may be because the pharmacists had less involvement at certain points of CVD interventions and experienced gaps in their commitments or skills. Such gaps indicate the need for increased focus on improving the level of counseling and involvement of community pharmacists in CVD prevention and maintenance.

The current study highlighted the difficulties associated with providing CVD patient counseling and health promotion services in UAE community pharmacies. These difficulties were most commonly due to a lack of coordination among healthcare providers and inadequate time. Similar findings have been reported by other studies in Ethiopia (Sendekie and Netere, 2022), Malaysia (Sia et al., 2020), and Qatar (El Hajj et al., 2016). This indicates that these barriers are the most common across different contexts. Therefore, there is a need to mitigate their impact through problem-based attention, thereby improving community pharmacists’ involvement in CVD prevention and management.

Based on the analysis of the current participants’ demographic characteristics, community pharmacists in the UAE who have more professional experience, see fewer patients per day, and have received training on how to manage and prevent CVD have higher perception and practice scores regarding CVD pharmaceutical care services. It could be reasoned that busy pharmacies have more clients, meaning that the pharmacists are unable to adequately offer counseling and may prefer to adhere to conventional methods of dispensing medicine to mitigate their workload. Therefore, community pharmacists should serve an ideal number of patients per day, which would enable them to offer effective services to all patients, including improved counseling and pharmaceutical services.

This study has several limitations. We used a cross-sectional design, which means we cannot draw conclusions regarding the causality of the regression model findings. Thus, the results cannot be generalized, and there is a need for further longitudinal research. We also used self-report questionnaires, meaning that the results could have been affected by social desirability and recall bias. Situations can occur wherein the sample size actually achieved is not enough to meet the sample size as calculated in advance (Supplementary Material). Nonetheless, these findings are robust due to the large number of community pharmacists included in the sample and their high response rate.

## 5 Conclusion

Pharmacy professionals working in community pharmacies in the UAE provide pharmaceutical care to patients to help manage and prevent CVD. Most participants demonstrated good perceptions and attitudes toward CVD prevention and management strategies. However, they also showed low levels of involvement in other healthcare services, such as screening and measuring patients’ weight, glucose levels, and blood pressure, monitoring treatment responses, maintaining medical records, and reviewing medication refill histories. These activities should be encouraged among UAE community pharmacists to ensure the provision of high-quality patient care. The provision of ongoing training and proper guidelines will assist pharmacy professionals in their ability to provide CVD screening, lifestyle modification advice, and prevention and treatment counseling by improving their confidence and involvement in caring for patients with CVD. For instance, a community pharmacist who receives training on the latest advancements in cardiovascular care could identify potential risks in a patient’s medication regimen and recommend adjustments using evidence-based guidelines. Through regular monitoring and counseling on lifestyle modifications, a pharmacist can assist a patient in achieving improved blood pressure and cholesterol levels, leading to increased patient confidence and improved outcomes.

## Data Availability

The original contributions presented in the study are included in the article/Supplementary Material, further inquiries can be directed to the corresponding authors.
