# Supplementary material for: Community pharmacists’ perspectives on cardiovascular disease pharmaceutical care in the United Arab Emirates: a questionnaire survey-based analysis
Source: Front Pharmacol. 2023 Sep 5;14:1237717. doi: 10.3389/fphar.2023.1237717 (PMC10509554; doi:10.3389/fphar.2023.1237717)
Supplement: Supplementary file 1 [file DataSheet1.PDF]

Conditions for not meeting a pre-calculated sample size in the current Study.

Situations can occur wherein the sample size actually achieved is not enough to meet the sample size as calculated in advance. Such an outcome may be due to several factors, as in the commonly occurring scenarios outlined below:

1. Difficulties in the recruitment phase: Even when meticulous plans have been made, the researchers may have difficulty in recruiting participants, e.g., because of a lack of time, insufficient interest from potential participants, and a lack of access to the target population, among other factors.

2. Limited budgets: Research tends to be a costly endeavor, and there may be limited funding available; this can have an impact on whether the study can recruit the required number of participants.

3. Limited time: There may be a strict timeline for the research, and it may not be possible to gather the desired sample size within the time given. This can force researchers to make do with smaller sample sizes.

4. Data quality issues: A study may face data quality issues, including incomplete or even missing data. Faced with such a situation, the researcher may be forced to leave out incomplete data, causing the sample size to shrink.

5. Ethical considerations: A study may be subject to ethical constraints that constrain it from recruiting enough participants. For example, research on vulnerable populations is likely to have limits on the participant number for reasons of well-being and safety.

6. Unpredictable circumstances: The progress of the research can be affected by unforeseen events that lie outside the researchers' control; this can significantly diminish the final sample size.

7. Requirements for statistical power: Under certain circumstances, the actual obtained sample size may be considered sufficient by the researcher to permit the necessary statistical power and meet the goals of the study.

In the event of any of the above, the researchers should note why they have chosen a smaller sample size and perform the results interpretation under consideration of the possible implications that the smaller sample size will have on the generalizability and validity of the research.
